# Transparency in Coverage Data and Variation in Prices for Common Health Care Services

**DOI:** 10.1001/jamahealthforum.2023.3663

**Published:** 2023-10-27

**Authors:** Benjamin L. Chartock, Kosali Simon, Christopher M. Whaley

**Affiliations:** 1 Department of Economics, Bentley University, Waltham, Massachusetts; 2 The Paul H. O’Neill School of Public and Environmental Affairs, Indiana University, Bloomington; 3 Department of Health Services, Policy and Practice, Brown University School of Public Health, Providence, Rhode Island

## Abstract

This cross-sectional study describes the health care prices publicly posted by Humana and the price variations by geography, service, and other factors.

## Introduction

Over half of the US population receives health insurance from private insurers, and prices are negotiated rather than set administratively (eg, Medicare). This negotiation process contributes to a landscape in which private insurance prices are both higher than Medicare rates and highly variable.^1^ The private market lacked meaningful price transparency for patients and purchasers until the recent implementation of Hospital Price Transparency and Transparency in Coverage (TiC) rules.^2^ Lack of transparency limits the ability of regulators to monitor prices and of employers, patients, and purchasers to impose market discipline on prices. We examined TiC price data for common services from Humana, a large national insurer, and highlighted use cases of such novel data for future research. New TiC payer data are released each month by all payers. Informed health care consumerism is a potential lever for managing costs and improving patient satisfaction.

## Methods

We obtained October 2022 TiC data from Humana’s public-facing portal and downloaded data in batches (Python Software).^3^ Indiana University Institutional Review Board deemed this cross-sectional study exempt from ethics review and informed consent because it was not human participant research.

Humana rates were chosen because of its largely national coverage of clinicians and facilities and our ability to speedily parse the data files. While mostly a provider of Medicare Advantage benefits, Humana covers approximately 1 million individuals with commercial insurance.^4^ We restricted analyses to in-network clinicians and facilities and used the mean posted price when the data included multiple contracted rates for the same procedure and clinician or facility within the same network.

We focused on 7 procedures, including more shoppable codes (computed tomography [CT] scan of head or brain without contrast) and less shoppable codes (high-severity emergency department [ED] visit). A key challenge was that TiC data reported rates for clinicians and facilities regardless of whether they actually performed a given service. To identify those who performed the selected services, we used both 2019 100% Medicare fee-for-service claims data and commercial claims data from the RAND hospital price transparency project^5^ to match clinicians and facilities who performed these services by their National Provider Identifiers. We analyzed distributional differences in prices (mean, median, and percentiles) and coefficients of variation. Data analysis was performed using Stata 17.0 (StataCorp LLC).

## Results

The Table presents descriptive characteristics of the study sample and price variation. The number of clinicians and facilities with Humana prices ranged from 4192 for hip arthroplasty to 189 471 for established patient office visit. Coefficients of variation were similar for both more and less shoppable services (0.51 for CT of head or brain without contrast; 0.53 for high-severity ED visit).

**Table.** Descriptive Characteristics of Study Sample

Health care service	No. of clinicians and facilities with Humana prices	Mean (median) [IQR] price, $	Ratio of 25th-75th percentiles	Coefficient of variation
Established patient office visit	189 471	99 (88) [69-114]	1.65	0.46
High-severity ED visit	16 757	268 (226) [169-320]	1.89	0.53
Colonoscopy	5714	470 (417) [348-528]	1.52	0.44
Lipid panel	24 972	19 (15) [12-21]	1.75	0.63
Lower-extremity MRI	6942	388 (333) [251-456]	1.82	0.55
Hip arthroplasty	4192	1735 (1498) [1231-1930]	1.57	0.47
CT of head or brain without contrast	6649	194 (164) [132-218]	1.65	0.51

Abbreviations: CT, computed tomography; ED, emergency department; MRI, magnetic resonance imaging.

The Figure maps the variation in prices for established patient office visits across US counties. The mean (IQR) county-level price was $86 ($69-$93). Generally, mean county-level prices were lowest in the central US and Florida. Prices were higher in the upper-Midwest and Southeast. Importantly, many higher-priced counties bordered lower-priced counties. Similar geographic patterns were observed for other procedures.

**Figure.**
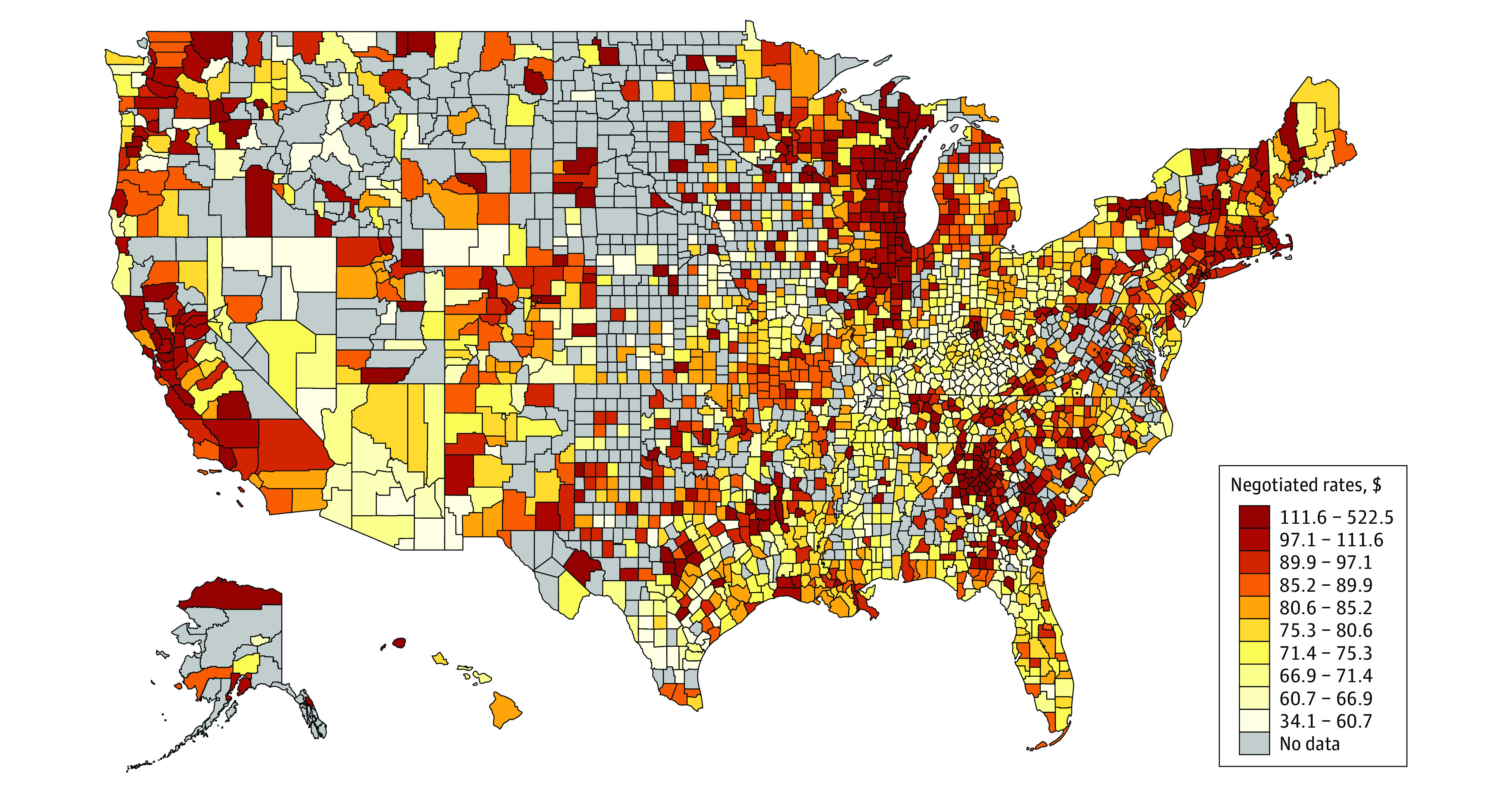
Negotiated Rates for Established Patient Office Visit

## Discussion

This study revealed how novel data can inform policies that improve the efficiency of the US health care system. The study was limited to a single insurer and 7 procedures; however, it opens the door to using TiC data in other, broader settings.

Future work may examine the underlying causes of price variation in health care, as it is unclear whether prices are meaningfully associated with value as in nearly every market, or whether prices reflect imbalances in market power and negotiation leverage. If price variation reflects clinical or perceived quality variation, purchasers and policymakers need to find balance between receiving higher-quality care and spending financial resources elsewhere. However, if price variation is driven by consolidation or anticompetitive contracting, then regulators should design policies that ensure competitive health care markets. The factors determining price variation are likely in the middle of these 2 possibilities.

## Notes

Supplement.
Data Sharing Statement
