# Supplementary material for: Transparency in Coverage Data and Variation in Prices for Common Health Care Services
Source: JAMA Health Forum. 2023 Oct 27;4(10):e233663. doi: 10.1001/jamahealthforum.2023.3663 (PMC10611987; doi:10.1001/jamahealthforum.2023.3663)
Supplement: Supplement. — Data Sharing Statement [file jamahealthforum-e233663-s001.pdf]

Data Sharing Statement

Chartock. Transparency in Coverage Data and Variation in Prices for Common Health Care Services. *JAMA Health Forum*. Published October 27, 2023.
doi:10.1001/jamahealthforum.2023.3663

Data
Data available: No

Additional Information
Explanation for why data not available: Some of the raw data used to merge with the publicly available data is under restricted use agreement with RAND.
